# Supplementary material for: The interactome of intact mitochondria by cross-linking mass spectrometry provides evidence for coexisting respiratory supercomplexes
Source: Mol Cell Proteomics. 2017 Dec 8;17(2):216–32. doi: 10.1074/mcp.RA117.000470 (PMC5795388; doi:10.1074/mcp.RA117.000470)
Supplement: Supplemental Data [file supp_RA117.000470_133922_0_supp_23978_fzffwf.zip › spectra_annotation/mito_spectra_annotation/486-1-4-1-1-1.pdf]

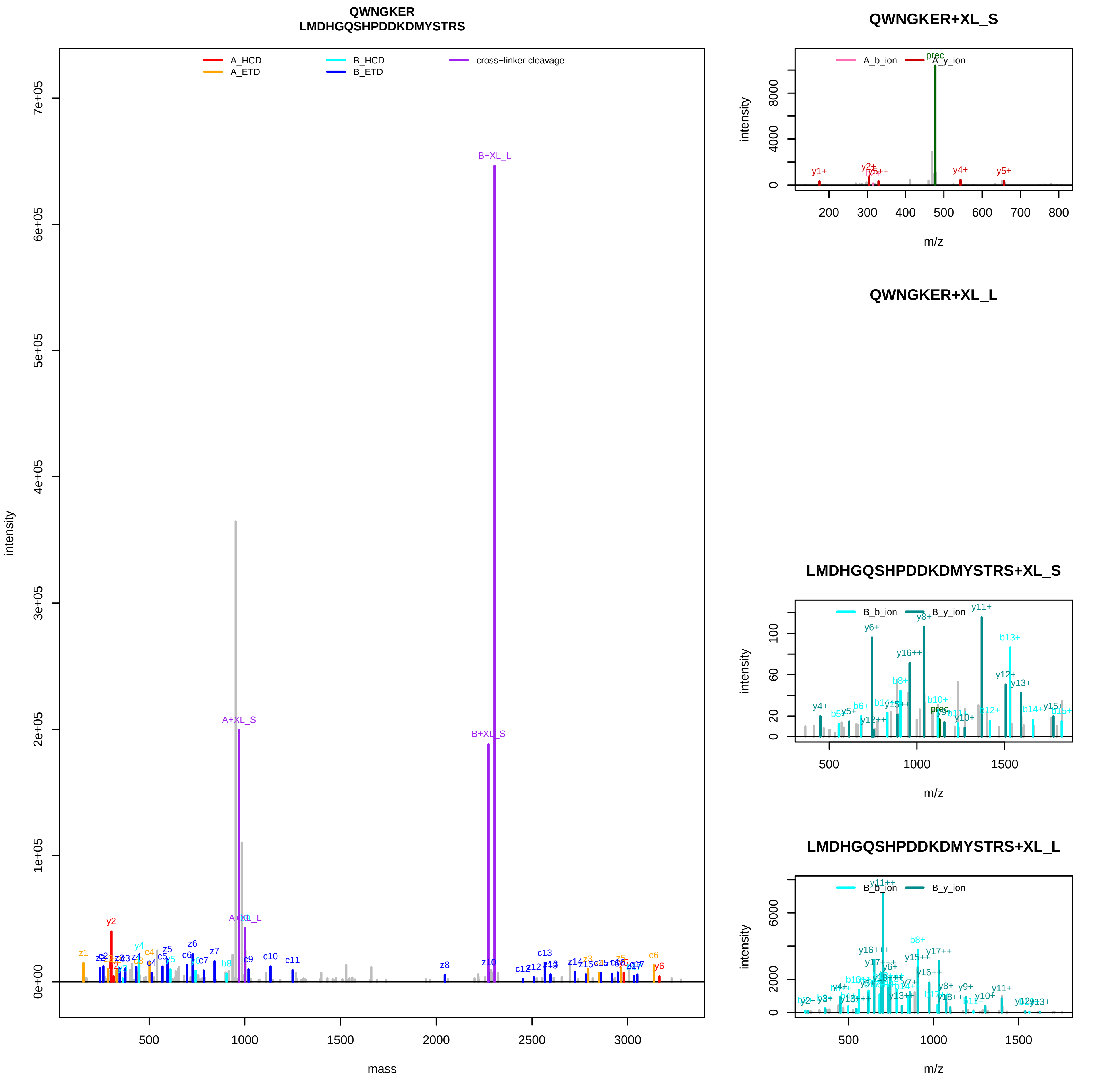
QWNGKER
LMDHGQSHPDDKDMYSTRS
A_HCD
A_ETD
B_HCD
B_ETD
cross−linker cleavage
intensity
mass
0e+00
1e+05
2e+05
3e+05
4e+05
5e+05
6e+05
7e+05
500
1000
1500
2000
2500
3000
B+XL_L
A+XL_S
B+XL_S
A+XL_L
y2
z1
z2
c2
z3
c3
z4
y4
c4
c5
z5
c6
z6
c7
z7
b8
c9
c10
c11
z8
z10
c12
z12
c13
z14
z15
c6
y6
QWNGKER+XL_S
A_b_ion
A_y_ion
prec
intensity
m/z
y1+
y2+
y5++
y4+
y5+
QWNGKER+XL_L
LMDHGQSHPDDKDMYSTRS+XL_S
B_b_ion
B_y_ion
y11+
y8+
y6+
b13+
y16++
y12+
y13+
y4+
y15+
LMDHGQSHPDDKDMYSTRS+XL_L
y11++
b8+
y17++
y16+++
y15++
y9+
y11+
y10+
